# 4-{(4-Chloro­phen­yl)[4-(4-methyl­phen­yl)-1,2,3-selenadiazol-5-yl]meth­yl}-4,5,6,7-tetra­hydro-1,2,3-benzoselenadiazole

**DOI:** 10.1107/S160053681101751X

**Published:** 2011-05-14

**Authors:** J. Muthukumaran, M. Nishandhini, S. Chitra, S. Muthusubramanian, P. Manisankar, Suman Bhattacharya, R. Krishna, J. Jeyakanthan

**Affiliations:** a Centre for Bioinformatics, School of Life Sciences, Pondicherry University, Puducherry 605 014, India; b Department of Bioinformatics, Alagappa University, Karaikudi 630 003, India; c Department of Industrial Chemistry, Alagappa University, Karaikudi 630 003, India; d Department of Organic Chemistry, Madurai Kamaraj University, Madurai 625 021, India; e Department of Chemistry, Pondicherry University, Puducherry 605 014, India

## Abstract

In the title compound, C_22_H_19_ClN_4_Se_2_, the mean plane of the non-fused selenadiazole ring forms dihedral angles of 54.20 (16)° and 70.48 (11)°, respectively, with the essentially planar [maximum deviations of 0.025 (5) and 0.009 (2) Å, respectively] methyl­phenyl and chloro­phenyl substituents. The tetra­hydro-1,2,3-benzoselenadiazole group is disordered over two sets of sites with a refined occupancy ratio of 0.802 (5):0.198 (5). In the crystal, weak inter­molecular C—H⋯N inter­actions are observed.

## Related literature

For biological applications of 1,2,3-selenadiazole derivatives, see: Kuroda *et al.* (2001 ▶); El-Bahaie *et al.* (1990 ▶); El-Kashef *et al.* (1986 ▶); Plano *et al.* (2010 ▶); Padmavathi *et al.* (2002 ▶). For the structures of mono and bis-1,2,3-selenadiazole derivatives, see: Marx *et al.* (2008 ▶); Boag *et al.* (2010 ▶). For ring puckering analysis, see: Cremer & Pople (1975 ▶).
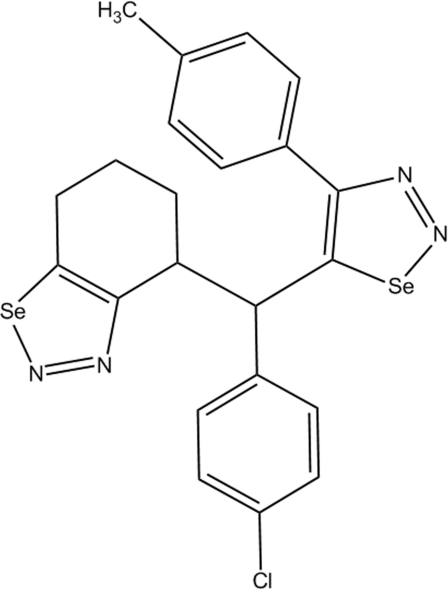


## Experimental

### 

#### Crystal data

C_22_H_19_ClN_4_Se_2_
*M* *_r_* = 532.78
Monoclinic, 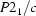

*a* = 9.7226 (18) Å
*b* = 12.969 (4) Å
*c* = 17.690 (3) Å
β = 100.959 (19)°
*V* = 2189.9 (8) Å^3^
*Z* = 4
Mo *K*α radiation
μ = 3.52 mm^−1^
*T* = 293 K
0.4 × 0.3 × 0.2 mm

#### Data collection

Oxford Diffraction Xcalibur Eos diffractometer
Absorption correction: multi-scan (*CrysAlis PRO*; Oxford Diffraction, 2009 ▶) *T*_min_ = 0.516, *T*_max_ = 1.000
8795 measured reflections
3849 independent reflections
2590 reflections with *I* > 2σ(*I*)
*R*_int_ = 0.041

#### Refinement

*R*[*F* ^2^ > 2σ(*F* ^2^)] = 0.051
*wR*(*F* ^2^) = 0.138
*S* = 1.04
3849 reflections
255 parameters
293 restraints
H-atom parameters constrained
Δρ_max_ = 0.70 e Å^−3^
Δρ_min_ = −0.68 e Å^−3^

### 

Data collection: *CrysAlis CCD* (Oxford Diffraction, 2009 ▶); cell refinement: *CrysAlis RED* (Oxford Diffraction, 2009 ▶); data reduction: *CrysAlis RED*; program(s) used to solve structure: *SHELXS97* (Sheldrick, 2008 ▶); program(s) used to refine structure: *SHELXL97* (Sheldrick, 2008 ▶); molecular graphics: *ORTEP-3 for Windows* (Farrugia, 1997 ▶) and *PLATON* (Spek, 2009 ▶); software used to prepare material for publication: *PLATON*.

## Supplementary Material

Crystal structure: contains datablocks I, global. DOI: 10.1107/S160053681101751X/lh5230sup1.cif

Structure factors: contains datablocks I. DOI: 10.1107/S160053681101751X/lh5230Isup2.hkl

Additional supplementary materials:  crystallographic information; 3D view; checkCIF report

## Figures and Tables

**Table 1:** Hydrogen-bond geometry (Å, °)

*D*—H⋯*A*	*D*—H	H⋯*A*	*D*⋯*A*	*D*—H⋯*A*
C6*A*—H6*A*⋯N2*A*^i^	0.98	2.57	3.517 (16)	164
C10—H10⋯N4^ii^	0.93	2.61	3.467 (7)	153
C12—H12⋯N3^iii^	0.93	2.61	3.470 (7)	153

Symmetry codes: (i) 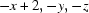; (ii) 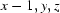; (iii) 
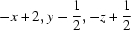
.

**Fig. 1.**
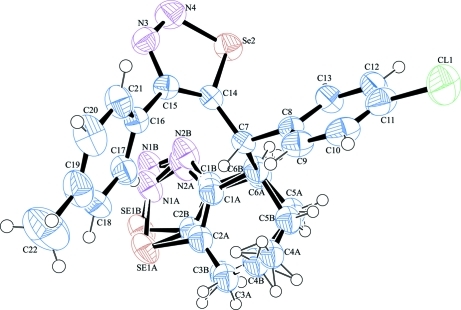
The molecular structure of (I), showing displacement ellipsoids drawn at the 50% probability level. The disorder components are shown.

**Fig. 2.**
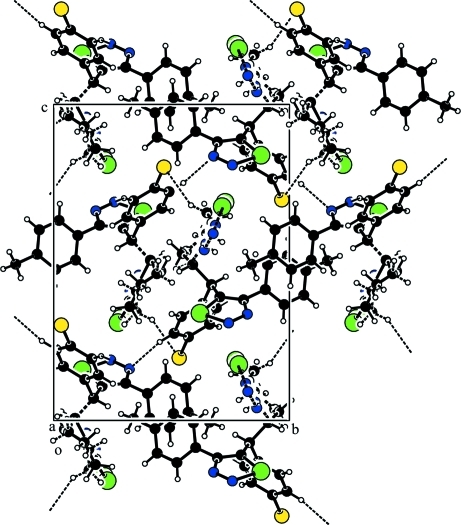
The crystal packing of (I) showing intermolecular interactions as dashed lines.

## supplementary crystallographic information

### Comment

Selenium containing compounds like 1,2,3-selenadiazole are of increasing interest because of their unique chemical properties and have several important biological applications such as anti-fungal (Kuroda *et al.*, 2001), anti-bacterial (El-Kashef *et al.*,1986), anti-microbial (El-Bahaie *et al.*, 1990), anti-cancer (Plano *et al.*, 2010) and insecticidal (Padmavathi *et al.*, 2002) activities. In view of the growing importances of selenium containing compounds, we present herein the single-crystal structure of the title compound (I).

Some mono and bis-1,2,3-selenadiazole derivatives are already reported in the literature (Marx *et al.*, 2008; Boag *et al.*, 2010). The molecular structure of the title compound is shown in Fig. 1. The puckering analysis (Cremer & Pople, 1975) for the atoms C1A/C2A/C3A/C4A/C5A/C6A (belonging to the major component of disorder) of the 4,5,6,7-tetrahydrobenzo[*d*][1,2,3]selenadiazole group in (I) adopt an envelope conformation (E form) with puckering parameters of Q = 0.536 (8) Å, θ = 56.1 (8)° and Φ = 239.0 (10)°. The tetrahydro-1,2,3-benzoselenadiazole group is disordered with the refined site-occupancy ratios 0.802 (5):0.198 (5). The hetrocyclic ring (Se2/C14/C15/N3/N4) makes dihedral angles of 54.20 (16)° and 70.48 (11)°, respectively with the essentially planar atoms C16-C22 [maximum deviation 0.025 (5) Å for C16] and atoms C8-C13/Cl1 [maximum deviation of 0.009 (2)Å for Cl1]. The crystal packing (Fig. 2) is stabilized by weak intermolecular C—H···N interactions.

### Experimental

A mixture of 2-[1-(4-chloro-phenyl)-3-(4-methylphenyl)-3-oxopropyl]-1-cyclohexanone (1 mmol) and semicarbazide hydro-chloride (2.5 mmol) in ethanol (10 mL) was heated under reflux on a water bath for 3 h. After completion of the reaction as evident from TLC, the mixture was poured into ice-water (50 ml) and the resulting semicarbazone solid was filtered off. Then, a mixture of semicarbazone and SeO_2_ (3 mmol) in tetrahydrofuran (THF) (10 ml) were refluxed on a water bath for 30 min. After completion of the reaction as monitored by TLC, the reaction mixture was filtered to remove selenium powder, the filtrate was concentrated under vacuum, and the residue was subjected to column chromatography using petroleum ether/ethyl acetate mixture (95:5; *v*/*v*) as eluent to afford the pure product. X-ray quality crystals were grown from a solution of the title compound in a 3:1 mixture of dichloromethane:ethylacetate in a partially closed 5ml glass vial over 7-8 days.

### Refinement

All hydrogen atoms were placed in calculated positions, with C—H = 0.93 and included in the final cycles of refinement using a riding model with *U*_iso_(H) = 1.2 *U*_eq_(C). The atoms of the tetrahydro-1,2,3-benzoselenadiazole group are disordered with the refined site-occupancy ratios 0.802 (5):0.198 (5). The DFIX, SIMU, DELU and EADP commands in SHELXL (Sheldrick, 2008) were used to model the disorder.

### Crystal data

**Table:**

C_22_H_19_ClN_4_Se_2_	*F*(000) = 1056
*M**_r_* = 532.78	*D*_x_ = 1.616 Mg m^−^^3^
Monoclinic, *P*2_1_/*c*	Mo *K*α radiation, λ = 0.71073 Å
Hall symbol: -P 2ybc	Cell parameters from 2877 reflections
*a* = 9.7226 (18) Å	θ = 2.7–29.4°
*b* = 12.969 (4) Å	µ = 3.52 mm^−^^1^
*c* = 17.690 (3) Å	*T* = 293 K
β = 100.959 (19)°	Block, blue
*V* = 2189.9 (8) Å^3^	0.4 × 0.3 × 0.2 mm
*Z* = 4	

### Data collection

**Table:**

Oxford Diffraction Xcalibur Eos diffractometer	3849 independent reflections
Radiation source: fine-focus sealed tube	2590 reflections with *I* > 2σ(*I*)
graphite	*R*_int_ = 0.041
Detector resolution: 15.9821 pixels mm^-1^	θ_max_ = 25.0°, θ_min_ = 2.7°
ω scans	*h* = −11→11
Absorption correction: multi-scan (*CrysAlis PRO*; Oxford Diffraction, 2009)	*k* = −15→13
*T*_min_ = 0.516, *T*_max_ = 1.000	*l* = −20→21
8795 measured reflections	

### Refinement

**Table:**

Refinement on *F*^2^	Primary atom site location: structure-invariant direct methods
Least-squares matrix: full	Secondary atom site location: difference Fourier map
*R*[*F*^2^ > 2σ(*F*^2^)] = 0.051	Hydrogen site location: inferred from neighbouring sites
*wR*(*F*^2^) = 0.138	H-atom parameters constrained
*S* = 1.04	*w* = 1/[σ^2^(*F*_o_^2^) + (0.0596*P*)^2^ + 0.3633*P*] where *P* = (*F*_o_^2^ + 2*F*_c_^2^)/3
3849 reflections	(Δ/σ)_max_ = 0.001
255 parameters	Δρ_max_ = 0.70 e Å^−^^3^
293 restraints	Δρ_min_ = −0.68 e Å^−^^3^

### Special details

**Table:**

Geometry. All e.s.d.'s (except the e.s.d. in the dihedral angle between two l.s. planes) are estimated using the full covariance matrix. The cell e.s.d.'s are taken into account individually in the estimation of e.s.d.'s in distances, angles and torsion angles; correlations between e.s.d.'s in cell parameters are only used when they are defined by crystal symmetry. An approximate (isotropic) treatment of cell e.s.d.'s is used for estimating e.s.d.'s involving l.s. planes.
Refinement. Refinement of *F*^2^ against ALL reflections. The weighted *R*-factor *wR* and goodness of fit *S* are based on *F*^2^, conventional *R*-factors *R* are based on *F*, with *F* set to zero for negative *F*^2^. The threshold expression of *F*^2^ > σ(*F*^2^) is used only for calculating *R*-factors(gt) *etc*. and is not relevant to the choice of reflections for refinement. *R*-factors based on *F*^2^ are statistically about twice as large as those based on *F*, and *R*- factors based on ALL data will be even larger.

### Fractional atomic coordinates and isotropic or equivalent isotropic displacement parameters (Å^2^)

**Table:**

	*x*	*y*	*z*	*U*_iso_*/*U*_eq_	Occ. (<1)
Se2	1.09476 (5)	0.12061 (4)	0.16057 (3)	0.0585 (2)	
Se1A	0.8917 (3)	0.22707 (11)	−0.19037 (8)	0.0718 (5)	0.802 (5)
N1A	1.0459 (4)	0.1859 (6)	−0.1174 (3)	0.0651 (10)	0.802 (5)
N2A	1.0132 (5)	0.1456 (10)	−0.0585 (4)	0.0651 (10)	0.802 (5)
C1A	0.8710 (5)	0.1372 (9)	−0.0608 (4)	0.0550 (7)	0.802 (5)
C2A	0.7819 (4)	0.1715 (6)	−0.1252 (2)	0.0550 (7)	0.802 (5)
C3A	0.6210 (4)	0.1768 (5)	−0.1395 (3)	0.0550 (7)	0.802 (5)
H3A1	0.5816	0.1386	−0.1857	0.066*	0.802 (5)
H3A2	0.5902	0.2478	−0.1464	0.066*	0.802 (5)
C4A	0.5716 (6)	0.1296 (5)	−0.0694 (3)	0.0550 (7)	0.802 (5)
H4A1	0.4790	0.1003	−0.0862	0.066*	0.802 (5)
H4A2	0.5637	0.1841	−0.0330	0.066*	0.802 (5)
C5A	0.6695 (6)	0.0453 (5)	−0.0280 (5)	0.0550 (7)	0.802 (5)
H5A1	0.6280	0.0150	0.0125	0.066*	0.802 (5)
H5A2	0.6802	−0.0087	−0.0644	0.066*	0.802 (5)
C6A	0.8145 (7)	0.0897 (9)	0.0070 (3)	0.0550 (7)	0.802 (5)
H6A	0.8761	0.0329	0.0284	0.066*	0.802 (5)
Se1B	0.9566 (8)	0.2149 (5)	−0.1767 (3)	0.0718 (5)	0.198 (5)
N1B	1.0899 (15)	0.176 (3)	−0.0914 (11)	0.0651 (10)	0.198 (5)
N2B	1.0313 (16)	0.139 (5)	−0.0400 (17)	0.0651 (10)	0.198 (5)
C1B	0.8867 (16)	0.136 (3)	−0.0542 (15)	0.0550 (7)	0.198 (5)
C2B	0.8184 (11)	0.179 (2)	−0.1217 (9)	0.0550 (7)	0.198 (5)
C3B	0.6665 (17)	0.150 (2)	−0.1572 (10)	0.0550 (7)	0.198 (5)
H3B1	0.6636	0.0825	−0.1811	0.066*	0.198 (5)
H3B2	0.6265	0.1999	−0.1961	0.066*	0.198 (5)
C4B	0.584 (2)	0.1489 (19)	−0.0911 (12)	0.0550 (7)	0.198 (5)
H4B1	0.5922	0.2149	−0.0647	0.066*	0.198 (5)
H4B2	0.4856	0.1352	−0.1107	0.066*	0.198 (5)
C5B	0.649 (2)	0.063 (2)	−0.0358 (17)	0.0550 (7)	0.198 (5)
H5B1	0.5952	0.0559	0.0050	0.066*	0.198 (5)
H5B2	0.6416	−0.0022	−0.0637	0.066*	0.198 (5)
C6B	0.803 (2)	0.083 (3)	0.0002 (14)	0.0550 (7)	0.198 (5)
H6B	0.8495	0.0184	0.0203	0.066*	0.198 (5)
C7	0.8116 (4)	0.1691 (3)	0.0701 (2)	0.0381 (10)	
H7	0.7625	0.2297	0.0451	0.046*	
Cl1	0.46832 (17)	0.03231 (15)	0.30184 (9)	0.0924 (6)	
N3	1.1287 (4)	0.3213 (4)	0.1634 (2)	0.0590 (11)	
C8	0.7283 (4)	0.1319 (4)	0.1297 (2)	0.0397 (11)	
C14	0.9555 (4)	0.2050 (4)	0.1089 (2)	0.0398 (11)	
C15	0.9973 (4)	0.3053 (4)	0.1179 (2)	0.0430 (11)	
N4	1.1966 (4)	0.2421 (4)	0.1914 (3)	0.0694 (13)	
C12	0.6800 (5)	0.0107 (4)	0.2241 (3)	0.0568 (13)	
H12	0.7008	−0.0501	0.2517	0.068*	
C16	0.9173 (5)	0.3976 (4)	0.0856 (2)	0.0431 (11)	
C19	0.7582 (6)	0.5702 (5)	0.0261 (4)	0.0690 (16)	
C9	0.6174 (5)	0.1898 (4)	0.1437 (3)	0.0525 (13)	
H9	0.5960	0.2510	0.1166	0.063*	
C11	0.5706 (5)	0.0717 (5)	0.2355 (3)	0.0596 (15)	
C10	0.5379 (5)	0.1612 (5)	0.1956 (3)	0.0563 (14)	
H10	0.4630	0.2016	0.2037	0.068*	
C13	0.7581 (5)	0.0413 (4)	0.1711 (2)	0.0507 (12)	
H13	0.8325	0.0004	0.1629	0.061*	
C17	0.8653 (6)	0.4074 (4)	0.0074 (3)	0.0575 (14)	
H17	0.8840	0.3561	−0.0259	0.069*	
C18	0.7867 (6)	0.4917 (4)	−0.0218 (3)	0.0696 (16)	
H18	0.7521	0.4959	−0.0745	0.084*	
C21	0.8923 (6)	0.4772 (4)	0.1331 (3)	0.0576 (13)	
H21	0.9291	0.4741	0.1855	0.069*	
C20	0.8137 (6)	0.5610 (4)	0.1034 (3)	0.0709 (16)	
H20	0.7975	0.6132	0.1367	0.085*	
C22	0.6694 (7)	0.6612 (6)	−0.0055 (4)	0.115 (3)	
H22A	0.6222	0.6878	0.0333	0.173*	
H22B	0.6014	0.6399	−0.0495	0.173*	
H22C	0.7280	0.7139	−0.0206	0.173*	

### Atomic displacement parameters (Å^2^)

**Table:**

	*U*^11^	*U*^22^	*U*^33^	*U*^12^	*U*^13^	*U*^23^
Se2	0.0497 (3)	0.0567 (4)	0.0679 (4)	0.0162 (3)	0.0084 (3)	0.0100 (3)
Se1A	0.0962 (12)	0.0770 (6)	0.0489 (5)	0.0015 (7)	0.0308 (7)	0.0137 (4)
N1A	0.077 (2)	0.060 (3)	0.069 (4)	0.018 (3)	0.038 (2)	0.015 (3)
N2A	0.077 (2)	0.060 (3)	0.069 (4)	0.018 (3)	0.038 (2)	0.015 (3)
C1A	0.0730 (15)	0.0516 (16)	0.0431 (13)	−0.0072 (14)	0.0177 (12)	−0.0051 (10)
C2A	0.0730 (15)	0.0516 (16)	0.0431 (13)	−0.0072 (14)	0.0177 (12)	−0.0051 (10)
C3A	0.0730 (15)	0.0516 (16)	0.0431 (13)	−0.0072 (14)	0.0177 (12)	−0.0051 (10)
C4A	0.0730 (15)	0.0516 (16)	0.0431 (13)	−0.0072 (14)	0.0177 (12)	−0.0051 (10)
C5A	0.0730 (15)	0.0516 (16)	0.0431 (13)	−0.0072 (14)	0.0177 (12)	−0.0051 (10)
C6A	0.0730 (15)	0.0516 (16)	0.0431 (13)	−0.0072 (14)	0.0177 (12)	−0.0051 (10)
Se1B	0.0962 (12)	0.0770 (6)	0.0489 (5)	0.0015 (7)	0.0308 (7)	0.0137 (4)
N1B	0.077 (2)	0.060 (3)	0.069 (4)	0.018 (3)	0.038 (2)	0.015 (3)
N2B	0.077 (2)	0.060 (3)	0.069 (4)	0.018 (3)	0.038 (2)	0.015 (3)
C1B	0.0730 (15)	0.0516 (16)	0.0431 (13)	−0.0072 (14)	0.0177 (12)	−0.0051 (10)
C2B	0.0730 (15)	0.0516 (16)	0.0431 (13)	−0.0072 (14)	0.0177 (12)	−0.0051 (10)
C3B	0.0730 (15)	0.0516 (16)	0.0431 (13)	−0.0072 (14)	0.0177 (12)	−0.0051 (10)
C4B	0.0730 (15)	0.0516 (16)	0.0431 (13)	−0.0072 (14)	0.0177 (12)	−0.0051 (10)
C5B	0.0730 (15)	0.0516 (16)	0.0431 (13)	−0.0072 (14)	0.0177 (12)	−0.0051 (10)
C6B	0.0730 (15)	0.0516 (16)	0.0431 (13)	−0.0072 (14)	0.0177 (12)	−0.0051 (10)
C7	0.041 (2)	0.038 (3)	0.035 (2)	0.007 (2)	0.009 (2)	0.0016 (19)
Cl1	0.0783 (10)	0.1362 (16)	0.0753 (9)	−0.0163 (10)	0.0468 (8)	−0.0029 (9)
N3	0.052 (2)	0.059 (3)	0.064 (3)	−0.003 (2)	0.004 (2)	−0.001 (2)
C8	0.034 (2)	0.047 (3)	0.038 (2)	0.004 (2)	0.0054 (19)	−0.004 (2)
C14	0.043 (2)	0.040 (3)	0.039 (2)	0.008 (2)	0.014 (2)	0.003 (2)
C15	0.036 (2)	0.048 (3)	0.045 (3)	0.006 (2)	0.010 (2)	−0.001 (2)
N4	0.045 (2)	0.085 (4)	0.076 (3)	0.006 (3)	0.006 (2)	0.008 (3)
C12	0.052 (3)	0.064 (4)	0.056 (3)	0.007 (3)	0.014 (3)	0.015 (3)
C16	0.041 (2)	0.043 (3)	0.048 (3)	0.003 (2)	0.013 (2)	0.002 (2)
C19	0.060 (3)	0.057 (4)	0.094 (4)	0.016 (3)	0.027 (3)	0.020 (3)
C9	0.042 (3)	0.063 (4)	0.052 (3)	0.011 (3)	0.007 (2)	−0.005 (2)
C11	0.042 (3)	0.092 (5)	0.047 (3)	−0.007 (3)	0.014 (2)	−0.009 (3)
C10	0.038 (3)	0.075 (4)	0.057 (3)	0.011 (3)	0.011 (2)	−0.014 (3)
C13	0.044 (3)	0.058 (3)	0.053 (3)	0.015 (3)	0.017 (2)	0.008 (2)
C17	0.070 (3)	0.048 (3)	0.055 (3)	0.000 (3)	0.014 (3)	0.005 (2)
C18	0.079 (4)	0.058 (4)	0.065 (3)	0.006 (4)	−0.003 (3)	0.020 (3)
C21	0.068 (3)	0.050 (3)	0.059 (3)	0.004 (3)	0.025 (3)	0.004 (3)
C20	0.085 (4)	0.058 (4)	0.082 (4)	0.021 (3)	0.046 (3)	0.004 (3)
C22	0.107 (6)	0.104 (6)	0.135 (6)	0.053 (5)	0.021 (5)	0.038 (5)

### Geometric parameters (Å, °)

**Table:**

Se2—C14	1.843 (4)	C5B—H5B2	0.9700
Se2—N4	1.886 (5)	C6B—C7	1.66 (4)
Se1A—C2A	1.8585 (10)	C6B—H6B	0.9800
Se1A—N1A	1.8605 (11)	C7—C14	1.510 (6)
N1A—N2A	1.2601 (10)	C7—C8	1.526 (6)
N2A—C1A	1.3790 (10)	C7—H7	0.9800
C1A—C2A	1.3688 (10)	Cl1—C11	1.753 (5)
C1A—C6A	1.5387 (10)	N3—N4	1.270 (6)
C2A—C3A	1.5385 (10)	N3—C15	1.390 (6)
C3A—C4A	1.5394 (10)	C8—C9	1.375 (6)
C3A—H3A1	0.9700	C8—C13	1.386 (6)
C3A—H3A2	0.9700	C14—C15	1.363 (6)
C4A—C5A	1.5396 (10)	C15—C16	1.482 (6)
C4A—H4A1	0.9700	C12—C11	1.371 (7)
C4A—H4A2	0.9700	C12—C13	1.372 (6)
C5A—C6A	1.5394 (10)	C12—H12	0.9300
C5A—H5A1	0.9700	C16—C21	1.381 (6)
C5A—H5A2	0.9700	C16—C17	1.385 (6)
C6A—C7	1.523 (11)	C19—C20	1.377 (8)
C6A—H6A	0.9800	C19—C18	1.387 (8)
Se1B—C2B	1.8597 (11)	C19—C22	1.506 (8)
Se1B—N1B	1.8602 (11)	C9—C10	1.360 (7)
N1B—N2B	1.2600 (10)	C9—H9	0.9300
N2B—C1B	1.3798 (10)	C11—C10	1.364 (7)
C1B—C2B	1.3698 (10)	C10—H10	0.9300
C1B—C6B	1.5397 (10)	C13—H13	0.9300
C2B—C3B	1.5398 (11)	C17—C18	1.377 (7)
C3B—C4B	1.5399 (10)	C17—H17	0.9300
C3B—H3B1	0.9700	C18—H18	0.9300
C3B—H3B2	0.9700	C21—C20	1.373 (7)
C4B—C5B	1.5398 (10)	C21—H21	0.9300
C4B—H4B1	0.9700	C20—H20	0.9300
C4B—H4B2	0.9700	C22—H22A	0.9600
C5B—C6B	1.5398 (11)	C22—H22B	0.9600
C5B—H5B1	0.9700	C22—H22C	0.9600
			
C14—Se2—N4	86.7 (2)	C5B—C6B—C7	109 (3)
C2A—Se1A—N1A	86.59 (18)	C1B—C6B—H6B	110.6
N2A—N1A—Se1A	113.4 (3)	C5B—C6B—H6B	110.6
N1A—N2A—C1A	114.7 (4)	C7—C6B—H6B	110.6
C2A—C1A—N2A	118.1 (4)	C14—C7—C6A	113.4 (4)
C2A—C1A—C6A	121.0 (4)	C14—C7—C8	110.6 (3)
N2A—C1A—C6A	120.9 (4)	C6A—C7—C8	112.3 (4)
C1A—C2A—C3A	127.9 (3)	C14—C7—C6B	117.2 (8)
C1A—C2A—Se1A	107.2 (3)	C8—C7—C6B	110.1 (7)
C3A—C2A—Se1A	124.6 (3)	C14—C7—H7	106.7
C2A—C3A—C4A	108.3 (3)	C6A—C7—H7	106.7
C2A—C3A—H3A1	110.0	C8—C7—H7	106.7
C4A—C3A—H3A1	110.0	C6B—C7—H7	104.9
C2A—C3A—H3A2	110.0	N4—N3—C15	117.2 (4)
C4A—C3A—H3A2	110.0	C9—C8—C13	117.4 (4)
H3A1—C3A—H3A2	108.4	C9—C8—C7	119.3 (4)
C3A—C4A—C5A	113.9 (5)	C13—C8—C7	123.3 (4)
C3A—C4A—H4A1	108.8	C15—C14—C7	125.3 (4)
C5A—C4A—H4A1	108.8	C15—C14—Se2	109.5 (3)
C3A—C4A—H4A2	108.8	C7—C14—Se2	124.9 (3)
C5A—C4A—H4A2	108.8	C14—C15—N3	115.6 (4)
H4A1—C4A—H4A2	107.7	C14—C15—C16	127.1 (4)
C6A—C5A—C4A	111.2 (5)	N3—C15—C16	117.4 (4)
C6A—C5A—H5A1	109.4	N3—N4—Se2	111.0 (3)
C4A—C5A—H5A1	109.4	C11—C12—C13	118.6 (5)
C6A—C5A—H5A2	109.4	C11—C12—H12	120.7
C4A—C5A—H5A2	109.4	C13—C12—H12	120.7
H5A1—C5A—H5A2	108.0	C21—C16—C17	117.7 (5)
C7—C6A—C1A	111.1 (8)	C21—C16—C15	120.7 (4)
C7—C6A—C5A	113.9 (7)	C17—C16—C15	121.6 (4)
C1A—C6A—C5A	105.8 (5)	C20—C19—C18	117.2 (5)
C7—C6A—H6A	108.7	C20—C19—C22	121.8 (6)
C1A—C6A—H6A	108.7	C18—C19—C22	121.0 (6)
C5A—C6A—H6A	108.7	C10—C9—C8	122.6 (5)
C2B—Se1B—N1B	88.3 (7)	C10—C9—H9	118.7
N2B—N1B—Se1B	110.4 (11)	C8—C9—H9	118.7
N1B—N2B—C1B	117.5 (14)	C10—C11—C12	121.7 (5)
C2B—C1B—N2B	117.3 (12)	C10—C11—Cl1	119.2 (4)
C2B—C1B—C6B	120.3 (12)	C12—C11—Cl1	119.0 (5)
N2B—C1B—C6B	122.3 (14)	C9—C10—C11	118.4 (5)
C1B—C2B—C3B	121.5 (15)	C9—C10—H10	120.8
C1B—C2B—Se1B	106.1 (9)	C11—C10—H10	120.8
C3B—C2B—Se1B	125.5 (11)	C12—C13—C8	121.2 (4)
C2B—C3B—C4B	106.9 (12)	C12—C13—H13	119.4
C2B—C3B—H3B1	110.3	C8—C13—H13	119.4
C4B—C3B—H3B1	110.3	C18—C17—C16	121.3 (5)
C2B—C3B—H3B2	110.3	C18—C17—H17	119.4
C4B—C3B—H3B2	110.3	C16—C17—H17	119.4
H3B1—C3B—H3B2	108.6	C17—C18—C19	121.0 (5)
C5B—C4B—C3B	106.3 (15)	C17—C18—H18	119.5
C5B—C4B—H4B1	110.5	C19—C18—H18	119.5
C3B—C4B—H4B1	110.5	C20—C21—C16	120.6 (5)
C5B—C4B—H4B2	110.5	C20—C21—H21	119.7
C3B—C4B—H4B2	110.5	C16—C21—H21	119.7
H4B1—C4B—H4B2	108.7	C21—C20—C19	122.2 (5)
C6B—C5B—C4B	112.9 (16)	C21—C20—H20	118.9
C6B—C5B—H5B1	109.0	C19—C20—H20	118.9
C4B—C5B—H5B1	109.0	C19—C22—H22A	109.5
C6B—C5B—H5B2	109.0	C19—C22—H22B	109.5
C4B—C5B—H5B2	109.0	H22A—C22—H22B	109.5
H5B1—C5B—H5B2	107.8	C19—C22—H22C	109.5
C1B—C6B—C5B	114.0 (14)	H22A—C22—H22C	109.5
C1B—C6B—C7	102 (3)	H22B—C22—H22C	109.5
			
C2A—Se1A—N1A—N2A	−2.8 (7)	C5B—C6B—C7—C6A	−177 (17)
Se1A—N1A—N2A—C1A	1.9 (13)	C1B—C6B—C7—C8	177.6 (12)
N1A—N2A—C1A—C2A	0.5 (17)	C5B—C6B—C7—C8	56.9 (16)
N1A—N2A—C1A—C6A	−179.9 (10)	C14—C7—C8—C9	109.2 (4)
N2A—C1A—C2A—C3A	−175.9 (10)	C6A—C7—C8—C9	−123.1 (5)
C6A—C1A—C2A—C3A	4.5 (16)	C6B—C7—C8—C9	−119.7 (11)
N2A—C1A—C2A—Se1A	−2.6 (14)	C14—C7—C8—C13	−71.1 (5)
C6A—C1A—C2A—Se1A	177.8 (9)	C6A—C7—C8—C13	56.7 (6)
N1A—Se1A—C2A—C1A	2.8 (7)	C6B—C7—C8—C13	60.0 (12)
N1A—Se1A—C2A—C3A	176.3 (7)	C6A—C7—C14—C15	127.4 (5)
C1A—C2A—C3A—C4A	−3.4 (12)	C8—C7—C14—C15	−105.5 (5)
Se1A—C2A—C3A—C4A	−175.6 (5)	C6B—C7—C14—C15	127.3 (10)
C2A—C3A—C4A—C5A	−29.7 (8)	C6A—C7—C14—Se2	−59.8 (5)
C3A—C4A—C5A—C6A	64.0 (7)	C8—C7—C14—Se2	67.3 (4)
C2A—C1A—C6A—C7	−97.8 (10)	C6B—C7—C14—Se2	−59.9 (10)
N2A—C1A—C6A—C7	82.7 (14)	N4—Se2—C14—C15	1.2 (3)
C2A—C1A—C6A—C5A	26.3 (14)	N4—Se2—C14—C7	−172.6 (4)
N2A—C1A—C6A—C5A	−153.3 (12)	C7—C14—C15—N3	172.5 (4)
C4A—C5A—C6A—C7	64.0 (7)	Se2—C14—C15—N3	−1.2 (5)
C4A—C5A—C6A—C1A	−58.3 (10)	C7—C14—C15—C16	−6.5 (7)
C2B—Se1B—N1B—N2B	5(3)	Se2—C14—C15—C16	179.8 (3)
Se1B—N1B—N2B—C1B	−2(5)	N4—N3—C15—C14	0.5 (6)
N1B—N2B—C1B—C2B	−3(7)	N4—N3—C15—C16	179.6 (4)
N1B—N2B—C1B—C6B	174 (4)	C15—N3—N4—Se2	0.5 (5)
N2B—C1B—C2B—C3B	158 (4)	C14—Se2—N4—N3	−1.0 (4)
C6B—C1B—C2B—C3B	−18 (6)	C14—C15—C16—C21	125.1 (5)
N2B—C1B—C2B—Se1B	6(5)	N3—C15—C16—C21	−53.8 (6)
C6B—C1B—C2B—Se1B	−170 (4)	C14—C15—C16—C17	−55.1 (7)
N1B—Se1B—C2B—C1B	−6(3)	N3—C15—C16—C17	126.0 (5)
N1B—Se1B—C2B—C3B	−157 (3)	C13—C8—C9—C10	−0.2 (7)
C1B—C2B—C3B—C4B	45 (4)	C7—C8—C9—C10	179.5 (4)
Se1B—C2B—C3B—C4B	−169 (2)	C13—C12—C11—C10	0.1 (8)
C2B—C3B—C4B—C5B	−64 (2)	C13—C12—C11—Cl1	179.0 (4)
C3B—C4B—C5B—C6B	63 (3)	C8—C9—C10—C11	0.5 (7)
C2B—C1B—C6B—C5B	12 (6)	C12—C11—C10—C9	−0.4 (8)
N2B—C1B—C6B—C5B	−164 (5)	Cl1—C11—C10—C9	−179.3 (4)
C2B—C1B—C6B—C7	−105 (4)	C11—C12—C13—C8	0.1 (7)
N2B—C1B—C6B—C7	79 (5)	C9—C8—C13—C12	−0.1 (7)
C4B—C5B—C6B—C1B	−36 (5)	C7—C8—C13—C12	−179.8 (4)
C4B—C5B—C6B—C7	77 (2)	C21—C16—C17—C18	−2.6 (7)
C1A—C6A—C7—C14	−65.9 (6)	C15—C16—C17—C18	177.6 (5)
C5A—C6A—C7—C14	174.8 (4)	C16—C17—C18—C19	0.8 (9)
C1A—C6A—C7—C8	167.9 (4)	C20—C19—C18—C17	1.1 (9)
C5A—C6A—C7—C8	48.6 (7)	C22—C19—C18—C17	−178.6 (6)
C1A—C6A—C7—C6B	112 (16)	C17—C16—C21—C20	2.6 (8)
C5A—C6A—C7—C6B	−7(16)	C15—C16—C21—C20	−177.6 (5)
C1B—C6B—C7—C14	−54.9 (18)	C16—C21—C20—C19	−0.8 (9)
C5B—C6B—C7—C14	−175.7 (10)	C18—C19—C20—C21	−1.1 (9)
C1B—C6B—C7—C6A	−57 (15)	C22—C19—C20—C21	178.6 (6)

### Hydrogen-bond geometry (Å, °)

**Table:**

*D*—H···*A*	*D*—H	H···*A*	*D*···*A*	*D*—H···*A*
C6A—H6A···N2A^i^	0.98	2.57	3.517 (16)	164
C10—H10···N4^ii^	0.93	2.61	3.467 (7)	153
C12—H12···N3^iii^	0.93	2.61	3.470 (7)	153

Symmetry codes: (i) −*x*+2, −*y*, −*z*; (ii) *x*−1, *y*, *z*; (iii) −*x*+2, *y*−1/2, −*z*+1/2.
